# Comorbid anxiety and depressive symptoms and the related factors among international medical students in China during COVID-19 pandemic: a cross-sectional study

**DOI:** 10.1186/s12888-023-04638-7

**Published:** 2023-03-14

**Authors:** Lulu Yuan, Lu Lu, Xuehang Wang, Min Qu, Yuqin Gao, Bochen Pan

**Affiliations:** 1 Center for Reproductive Medicine, Department of Obstetrics and Gynecology, Shengjing Hospital of China Medical University, Shenyang, China; 2 School and Hospital of Stomatology, China Medical University, Liaoning Provincial Key Laboratory of Oral Diseases, Shenyang, China; 3 China Medical University-The Queen’s University of Belfast Joint College, China Medical University, Shenyang, China; 4 International Education School, China Medical University, Shenyang, China

**Keywords:** COVID-19, International medical students, Comorbidity, Mental health, Anxiety and depressive symptoms, Perceived stress

## Abstract

**Background:**

The two most prevalent mental health conditions are anxiety and depression and they often coexist (comorbidity) in an individual aggravating the person’s psychological or medical conditions. College students suffered from anxiety and depressive symptoms during the COVID-19 pandemic, according to numerous studies. The lack of information on the comorbidity of anxiety and depression (CAD) among international medical students, however, makes it difficult to develop effective policies or strategies to support these students.

**Objective:**

The present research seeks to investigate the incidence of CAD among international medical students in China and to identify the variables that may be useful in predicting CAD.

**Method:**

A cross-sectional study was conducted at China Medical University in Shenyang, China, for international medical students during November 2020. A total of 519 international students provided information on their demographics, stress related to the COVID-19 pandemic, generalized anxiety disorder assessment (GAD-7), patient health questionnaire-9 (PHQ-9), simplified coping style questionnaire (SCSQ), perceived stress scale (PSS-10), the multidimensional scale of perceived social support (MSPSS), revised life orientation test (LOT-R), and resilience scale-14 (RS-14). To investigate the potential predictors of CAD, a chi-square test, a nonparametric test, and multinomial logistic regression analyses were carried out as appropriate.

**Results:**

The incidence of anxiety, depression, and CAD in the current study was 5.8%, 8.9%, and 22.7%, respectively. The predictors for students having symptoms of anxiety were observed to be the negative coping style (β = 0.662, OR = 1.938, CI:1.07–3.694) and perceived stress (β = 0.167, OR = 1.181, CI:1.076–1.297); the predictors for students having symptoms of depression were observed to be the COVID-19 pandemic-related stress (β = 0.323,OR = 1.382,CI:1.211–1.577), negative coping style (β = 0.693,OR = 2.000, CI:1.21–3.568), and perceived stress (β = 0.135,OR = 1.145,CI:1.050–1.248); whereas the predictors for students with CAD were observed to be staying up late (Yes VS No) (β = 1.028,OR = 2.794,CI:1.227–6.364), current place of residence (Other continents VS China) (β = -1.413, OR = 0.243,CI:0.065–0.910), COVID-19 pandemic-related stress (β = 0.371,OR = 1.450,CI:1.284–1.636), negative coping style (β = 1.092,OR = 2.979,CI:1.706–5.203), and perceived stress (β = 0.339,OR = 1.403,CI:1.289–1.527).

**Conclusion:**

Single anxiety and depressive symptoms were moderately prevalent among international medical students in China. However, CAD turned out to be the most prevalent mental health issue due to its relatively higher incidence. Negative coping style and perceived stress were the communal predictors of the three categories, whereas stress related to the COVID-19 pandemic was linked to both depression and CAD, and staying up late and in residential places were specific predictors for CAD. Study results suggest that COVID-19 pandemic-related stress was related to students’ CAD and depressive symptoms, and specific intervention measures with stress reduction, proper coping strategy, and a good lifestyle might be useful in improving the international students’ mental health status.

## Introduction

Particularly during the COVID-19 pandemic, anxiety and depression are commonly prevalent mental health disorders [1]. A higher prevalence of anxiety and depression was reported among adults [2], hospital staff [3], patients [4], COVID-19 survivors [5, 6], and the general population [7, 8] during the pandemic. The same may be true for medical students, because, in addition to the same stress experienced by the general population [1] and the students of other subjects [9], medical students may have to face more stress during the pandemic unique to their specialty features such as the interruption of clinical rotation and clerkship [10], which could make them more prone to develop mental health problems. Indeed, studies have demonstrated that anxiety and/or depression are highly prevalent in medical students [11–13], and similar results have been found in our recent study on international medical students [14]. In addition, a systematic review by Puthran [15] and Quek [16] showed alarmingly high prevalence of depression and anxiety respectively in medical students globally, compared to the general population, highlighting the importance of this research topic.

However, it is well known that anxiety and depression can co-occur in the same person, a condition often referred to as comorbid anxiety and depression (CAD). For example, one recent study has shown that over sixty percent of the participants with current anxiety or depressive disorder had a concurrent depression or anxiety disorder [17], and the same phenomenon also exists in students [18–20]. Anxiety and depression are known to be highly correlated, and they both exhibit a number of the same symptoms, including irritability, restlessness, poor concentration, irregular sleep patterns, and fatigue [21]. Currently, there is no consensus on the mode of relationship between anxiety and depressive disorders in CAD, and three hypotheses have been put forward to illuminate the relationship: monism, dualism, and ternary theory. Monism is the idea that anxiety and depression in CAD belong to the same disease, that is, some researchers believe that anxiety symptoms are a part or whole of depression [22, 23]; dualism is the idea that anxiety and depression are separate mental health disorders [24]; while the ternary theory proposes that the coexistence of anxiety and depression is a third disease distinctive from an anxiety disorder or depressive disorder [20]. Despite diverse ideas about the relationship between comorbid anxiety and depressive disorders, it is generally accepted that the adverse impact of CAD on individuals is more noticeable than the single anxiety or depressive disorders. For example, CAD would aggravate a person’s disease state and impair their response to treatment [21, 25], and, compared with anxiety or depression alone, CAD was generally characterized by longer symptom duration, more chronicity, longer time to initial remission, and more recurrence [17]. It was also known associated with functional, somatic, and other mental health problems such as less social activity, loneliness, chronic pain, cardiovascular disease, myocardial infarction, insomnia, obsessive–compulsive disorder, borderline personality disorder, and attempted suicide in comparison with single anxiety or depression [17]. Therefore, CAD may represent more severe psychopathology or pathophysiology in contrast to anxiety or depression alone and should be given special attention. However, to our knowledge, there have been few reports on CAD for international medical students up to date.

Similarly, studies are needed to explore the related or predictive factors for CAD because these factors may be different from (albeit related to) those for anxiety or depression alone. Previous studies have shown that anxiety and depression are related to demographic factors. For instance, age, gender, and education levels were all related to anxiety/depressive symptoms among students [13, 18, 20, 26, 27]. On the other hand, coping, which is defined as the cognitive attempts and behavioral adaption to cope with stressors [28], has been linked to mental health issues, and those individuals who have negative coping styles are more likely to experience adverse outcomes [29]. In addition, numerous research studies have demonstrated an association between perceived stress, which is the individual's self-assessment of the threat from stressors, and anxiety and depressive symptoms [30, 31]. Additionally, psychological resources like optimism, resilience, and social support might play some roles as well. Social support helps people develop their behavioral patterns, social cognition, and values because it is the material or moral support that others provide to them when they are stressed out or in a difficult situation [32]. A psychological resource that benefits a person's health is optimism, which is the human tendency to have optimistic expectations for the future [33]. Last but not least, resilience is the capacity of an individual to grow in the face of stressors or negative changes [34]. It has been demonstrated that these psychological tools positively help students who are anxious and depressed [30, 35–38]. It is crucial to investigate all these pertinent psychosocial factors when addressing stress and the mental health issues it can cause so that we can offer the students comprehensive psychological assistance to ease their CAD symptoms.

Recent studies have found that compared to students in other subjects, medical students experience higher levels of anxiety and depression [11, 19, 39]. Among medical students, the international medical students who study medicine in other countries instead of their own are deserving of even more special attention, because they may have issues of trans-cultural adaptation, residential separation from family, or the time differences for the online courses which demand quite an effort to catch up and sometimes can be stressful to them. We could foresee that these students' stress from the aforementioned sources might lead to mental health issues like CAD. But little research has been carried out to date to examine their functions in the mental well-being of international medical students, and we would like to fill the gap with our study. To that end, we are inspired to reprocess the data of our previous study [14], and divide our study population into four categories according to their mental health conditions, namely, healthy (free from anxiety or depressive symptoms), anxiety, depression, and CAD. We hypothesize that stress brought on by the COVID-19 pandemic, a negative coping style, and perceived stress are all directly correlated to (CAD), whereas a good coping style, perceived social support, optimism, and resilience are negatively correlated. The overall objective of this research is to investigate the incidence of CAD among international medical students in China before identifying any potential variables that might predict CAD.

## Methods

The current study is designed to be a cross-sectional study. Data were collected online at China Medical University during November 2020.

### Recruitment

International medical students who are currently enrolled at China Medical University and have internet access and can fully comprehend the survey's content in English were the study's participants. Participants were contacted by email with a link to access the English informed consent letter and online questionnaire (*N* = 1030). Only by clicking “Agree” on the informed consent form can the questionnaire be answered. If the respondent does not click “Agree” on the informed consent form, he/she is deemed to have refused to participate in the study and cannot answer the questionnaire. In the end, 550 students completed the questionnaire.

### Demographic data collection

The following were among the demographic data at the baseline: (1) General personal information: age, gender, academic background, residential place and style, current city-wide COVID-19 outbreak, smoking, consuming alcohol, exercising, staying up late, and Internet addiction; (2) stress associated to the COVID-19 pandemic: perception of the COVID-19 outbreak, worrying about oneself and family/friends/relatives contracting the illness, worrying about exam results, and concerns about not being able to finish studying.

### Assessment of anxiety, depression, and CAD

Anxiety was evaluated via the Generalized Anxiety Disorder Assessment(GAD-7) [40]. The GAD-7 score is calculated by assigning scores of 0, 1, 2, and 3, to the response categories of 'not at all', 'several days', 'more than half the days', and 'nearly every day', respectively, and adding together the scores for the seven questions. Scores of 5, 10, and 15 are taken as the cut-off points for mild, moderate and severe anxiety, respectively. In this study, participants with GAD-7 scores ≥ 5 indicated anxieties [41]. Depression was evaluated via the Patient Health Questionnaire-9 (PHQ-9) [42]. The PHQ-9 score is calculated by assigning scores of 0, 1, 2, and 3, to the response categories of 'not at all', 'several days', 'more than half the days', and 'nearly every day', respectively, and adding together the scores for the nine questions. Scores of 5, 10, 15, and 20 are taken as the cut-off points for mild, moderate, moderate-severe and severe depression, respectively. In this study, participants with PHQ-9 scores ≥ 5 were considered as having depression status [42]. A participant was considered suffering from CAD when both criteria for defining anxiety and depression were met. In this study, GAD-7 and PHQ-9 were chosen for measuring anxiety and depression, respectively, because they were the most widely used scales with good validity and reliability in different populations.

### Assessment of coping style, perceived stress, social support, optimism and resilience

In this study, five scales were used to measure coping style, perceived stress, social support, optimism and resilience, respectively. They were chosen because they showed good validity and reliability in different populations. The Simplified Coping Style Questionnaire (SCSQ) was employed to assess coping style [43]. The SCSQ is a 20-item scale with two domains, positive coping style and negative coping style, and answer to each item was scored on a 4-point scale (0–3). Positive coping strategies demonstrated a positive coping style, while negative coping strategies demonstrated a negative coping style. A relatively high domain score indicated an inclination for using the appropriate coping mechanism.

The perceived stress levels were evaluated via the 10-item version of the Perceived Stress Scale (PSS-10) [44]. On a 5-point scale, each item was scored. Higher scores imply a higher degree of perceived stress.

The Multidimensional Scale of Perceived Social Support (MSPSS) was employed for assessing the level of perceived social support among international medical students [45]. A higher score on the MSPSS, a 12-item scale with a 7-point rating system, indicated greater social support.

Optimism was evaluated via the Revised Life Orientation Test (LOT-R) [33]. It’s a ten items scale using a 5-point rating system. Three of the ten items were for optimism, three were for pessimism, and the remaining four items were fillers. A high score indicated a higher tendency for optimism.

The Resilience Scale-14 (RS-14) was employed for measuring resilience [46]. It contains 14 items using Likert's 7-level scoring method. Scores that were higher implied greater resilience.

### Operational definition

In the study, participants with only anxiety but not depressive symptoms were divided into the anxiety group; participants with only depressive but not anxiety symptoms were divided into the depression group; participants suffering from both anxiety and depressive symptoms were divided as comorbid anxiety and depression(CAD) group; participants with neither anxiety nor depressive symptoms were divided as a healthy group.

### Statistical analysis

Data analysis was executed by employing the Statistical Package for Social Sciences (SPSS 20.0 for Windows). The level of 0.05 was considered the significance level for all statistical tests (2-tailed). For every continuous variable, normality and homogeneity of variance were first tested. The distributions of the categories for anxiety, depression, and CAD in the categorical demographic variables were described via the chi-square test. Nonparametric tests were employed for exploring the correlation among the anxiety group, depression group, CAD group, and the continuous variables (age, Covid-19 pandemic-related stress, positive and negative coping styles, perceived stress, perceived social support, optimism, and resilience). To identify the predictors, multinomial logistic regression analyses were performed. To avoid over-fitting the logistic regression models, the variables having *P* < 0.2 in the Chi-square tests and nonparametric tests were added to the regression analysis [47]. Data presented in the regression models comprised the regression coefficient (β), OR, and its 95% CI.

## Results

### Characteristics of the study participants

In the present research, 550 questionnaires were obtained. Among them, 519 were deemed valid, resulting in a 94.36% effective response rate. There were 325 (62.6%), 30 (5.8%), 46 (8.9%), and 118 (22.7%) individuals in the healthy, anxious, depressed, and CAD groups, respectively. The participants’ demographic details in the four groups are presented in Table 1. Significant differences (*P* < 0.05) were discovered among the groups in terms of current residence, current city-wide outbreak, residence style, staying up late, and Internet addiction.

**Table 1:** Demographic characteristics of study participants (*n* = 519)

	***N (%)***	***Healthy (%)***	***Anxiety (%)***	***Depression (%)***	***CAD (%)***	***X***^***2***^	***P***
**Gender**							
male	276 (53.18)	166 (60.14)	16 (5.80)	31 (11.23)	63 (22.83)	-1.02	0.31
female	243 (46.82)	159 (65.44)	14 (5.76)	15 (6.17)	55 (22.63)		
**Educational background**							
Undergraduate	453 (87.28)	275 (60.71)	28(6.18)	44 (9.71)	106 (23.40)	6.48	0.09
Master’s	32 (6.17)	27 (84.38)	1 (3.12)	0 (0.00)	4 (12.50)		
Doctoral	27 (5.20)	18 (66.67)	1 (3.70)	1 (3.70)	7 (25.93)		
Trainees	7 (1.35)	5 (71.42)	0 (0.00)	1 (14.29)	1 (14.29)		
**Current place of residence**							
China	68 (13.10)	40 (58.82)	2 (2.94)	6 (8.82)	20 (29.42)	6.47	0.04^*^
Asia outside China	376 (72.45)	229 (60.90)	23 (6.12)	36 (9.57)	88 (23.41)		
Other continents	75 (14.45)	56 (74.67)	5 (6.67)	4 (5.33)	10 (13.33)		
**Current city-wide Outbreak**							
Yes	409 (78.80)	243 (59.41)	25 (6.11)	37 (9.05)	104 (25.43)	-3.06	< 0.01^**^
No	110 (21.20)	82 (74.55)	5 (4.54)	9 (8.18)	14 (12.73)		
**Residence style**							
Live alone	170 (32.76)	98 (57.65)	4 (2.35)	19 (11.18)	49 (28.82)	-2.09	0.04^*^
Live with family or friends	349 (67.24)	227 (65.04)	26 (7.45)	27 (7.74)	69 (19.77)		
**Smoking**							
Yes	23 (4.43)	12 (52.18)	0 (0.00)	3 (13.04)	8 (34.78)	-1.31	0.19
No	496 (95.57)	313 (63.11)	30 (6.04)	43 (8.67)	110 (22.18)		
**Drinking alcohol**							
Yes	21 (4.05)	11 (52.38)	1 (4.76)	3 (14.29)	6 (28.57)	-0.99	0.32
No	498 (95.95)	314 (63.05)	29 (5.83)	43 (8.63)	112 (22.49)		
**Exercise**							
Yes	460 (88.63)	291 (63.26)	27 (5.87)	41 (8.91)	101 (21.96)	1.01	0.31
No	59 (11.37)	34 (57.63)	3 (5.09)	5 (8.47)	17 (28.81)		
**Staying up late**							
Yes	370 (71.29)	211 (57.03)	19 (5.14)	36(9.73)	104 (28.10)	-4.64	< 0.001^***^
No	149 (28.71)	114 (76.51)	11 (7.38)	10 (6.71)	14 (9.40)		
**Addicted to the Internet**							
Yes	414 (79.77)	242 (58.45)	22 (5.31)	41 (9.91)	109 (26.33)	-4.24	< 0.001^***^
No	105 (20.23)	83 (79.05)	8 (7.62)	5 (4.76)	9 (8.57)		

Analysis was performed with X^2^ test

*N* number

*CAD* comorbid anxiety and depression

*P* < 0.05^*^, *P* < 0.01^**^, *P* < 0.01^***^

### Distributions of anxiety, depression and CAD in continuous variables

Table 2 summarized the distributions of healthy, anxiety, depression, and CAD in continuous variables which included age, Covid-19 pandemic-related stress, positive and negative coping styles, perceived stress, perceived social support, optimism, and resilience. Results revealed that, with the exception of age and a positive coping style, the distribution of the four groups varied considerably across all of the variables (*P* < 0.05). In addition, it was discovered that anxiety and depressive symptoms were inversely correlated with perceived social support, optimism, and resilience, whereas negative coping styles and perceived stress were positively correlated.

**Table 2:** Distributions of anxiety, depression and CAD in continuous variables (*n* = 519, Median (IQR))

	***Healthy*** ***(n* = *325)***	***Anxiety*** ***(n* = *30)***	***Depression*** ***(n* = *46)***	***CAD*** ***(n* = *118)***	***Z***	***P***
**Age**	22 (3)	22 (2.25)	22 (2.25)	22 (3)	6.46	0.09
**Covid-19 pandemic-related stress**	10 (4)	10 (3.25)	13 (3.25)	13.5 (5.25)	116.26	< 0.01
**Positive coping style**	1.75 (1.17)	1.92 (1.15)	1.67 (1.60)	1.67 (0.92)	3.12	0.37
**Negative coping style**	1.13 (1.25)	1.63 (0.59)	1.5 (0.78)	1.56 (0.75)	39.50	< 0.01
**Perceived stress**	15(6)	19 (5.25)	18 (4)	21.5 (6.25)	167.89	< 0.01
**PSSS-12**	67 (21.50)	69 (21.50)	61 (20.50)	57.5 (22.25)	22.63	< 0.01
**Optimism**	14 (5)	13.5 (4)	14 (4.50)	12.5 (4)	23.13	< 0.01
**Resilience**	84 (25)	85 (14.50)	81 (15.25)	76 (23.25)	34.00	< 0.01

Analysis was performed with nonparametric tests

*CAD* comorbid anxiety and depression

*IQR* inter-quartile range

### Predictors of anxiety, depression and CAD

Multinomial Logistic regression analysis was carried out for identifying the predictors of anxiety, depression, and CAD. The logistic regression analysis comprised of variables that were attributed to the four groups (healthy, anxiety, depression, and CAD), such as demographic factors (age, academic background, current and style of residence, current city-wide outbreak, smoking, staying up late, and Internet addiction), stress related to the Covid-19 pandemic, negative coping style, perceived stress, perceived social support, optimism, and resilience. Multicollinearity diagnostic tests revealed that there wasn’t any multicollinearity among the predictor variables. Logistic regression was carried out and the findings were illustrated in Table 3. As a result, negative coping style and perceived stress were observed to be the predictors of students with anxiety symptoms; Covid-19 pandemic-related stress, negative coping style, and perceived stress were observed to be the predictors of students with depressive symptoms; staying up late, residential place, Covid-19 pandemic-related stress, negative coping style, and perceived stress were observed to be the predictors of students with CAD.

**Table 3:** Results of Logistic regression analysis on students with anxiety symptoms, depressive symptoms and CAD

**Contents**	**Variables**	**β**	**S.E,**	**Wals**	***P***	**OR (95% CI)**
**Anxiety symptoms**	Negative coping style	0.66	0.33	4050	0.04	1.94 (1.02,3.69)
	Perceived stress	0.17	0.05	12.26	0.00	1.18 (1.08,1.30)
	Constant	-4.42	2.85	2.40	012	-
**Depressive symptoms**	Covid-19 pandemic-related stress	0.32	0.07	22.94	0.00	1.38 (1.21,1.59)
	Negative coping style	0.69	0.30	5.51	0.03	2.00 (1.12,3.57)
	Perceived stress	0.14	0.04	9.46	< 0.01	1.15 (1.05,1.25)
	Constant	-7.26	2.46	8.70	< 0.01	-
**CAD**	Stay up late (Yes ***VS*** No)	1.03	0.42	5.99	0.01	2.79 (1.23,6.36)
	Current place of residence					
	Asia outside China *VS* China	-0.15	0.51	0.09	0.76	0.86 (0.32,2.31)
	Other continents *VS* China	-1.41	0.67	4.41	0.04	0.24 (0.07,0.91)
	Covid-19 pandemic-related stress	0.37	0.06	36.15	< 0.01	1.45 (1.28,1.64)
	Negative coping style	1.09	0.28	14.73	< 0.01	2.98 (1.71,5.20)
	Perceived stress	0.34	0.04	61.23	< 0.01	1.40 (1.29,1.53)
	Constant	-13.42	2.14	39.53	< 0.01	-

*SE* standard error, *OR* odds ratio, *CI* confidence interval, *VS* versus

Percentile 95% CIs for ORs are defined using the values that mark the upper and lower 2.5% of OR value

## Discussion

### Principal results

#### The prevalence of anxiety, depression and CAD in international medical students

An important finding of this study with respect to the incidence of mental health issues in international medical students is that the rate of CAD (22.7%) is much higher than that of separate depressive symptoms (8.9%) and anxiety (5.8%). This finding is consistent with the data from a recent review article on summary findings of CAD in the Netherlands Study of Depression and Anxiety (NESDA) study, a longitudinal cohort study comprising 2981 adults from community, primary care, or mental health care services. In that study, it was demonstrated that comorbidity was the rule in participants having depressive and/or anxiety disorders: 68% of the participants having a depressive disorder met the criteria for a current comorbid anxiety disorder, whereas 63% of people who currently had an anxiety disorder also had a comorbid depressive disorder [17]. Similarly, in our study, students with current CAD accounted for 60.7% of all three conditions. Our finding is important because, on one hand, it may be indicative of the severity of the mental health problems in our students as CAD is generally considered more complicated and more severe than the other two single conditions. On the other hand, it may also indicate that despite the difference in the sample sources of participants, our students suffering from CAD may share some features of the mental disorders in other studies so that successful strategies for dealing with such conditions in other studies may be adopted or modified to help our students.

In addition, we found that the incidence of CAD in international medical students in our study was greater as compared to the medical students (14.3%) in the world prior to the pandemic [48] and students majoring in other subjects (18.3%) [49], suggesting the adverse pandemic influence and students’ specialty characteristics. We could speculate several reasons for the surprisingly high prevalence of CAD in our students. Firstly, students might suffer from fears, limited physical activity, loneliness, uncertainty, and insecurity due to the pandemic. Secondly, as international students, they might encounter more difficulties and problems than other students, like language and cultural differences [13]. Thirdly, due to the COVID-19 pandemic, a large number of international students (86.9% in our study) have been forced to remain in their homes or native countries and are only able to attend online courses, which may be challenging as a result of time differences or issues with internet accessibility. Finally, for medical students, clinical rotations and clerkships are essential components of medical education for students, but they are occasionally unavailable online. The challenges or problems mentioned above may make students fearful and anxious, which, if lasting for a long period of time, might worsen the mental state of the students, and make them feel low, sad, lack interest, and eventually lead to CAD. All in all, our results indicate that the COVID-19 pandemic had a substantial negative influence on the mental health of international medical students. Therefore, effort should be made to identify the major related factors, and proper intervention strategies should be taken to assist such students.

#### Predictors of anxiety, depression and CAD in international medical students

An interesting and important finding of our study with the predictors of mental health problems was that CAD had more predictors (5) than depressive symptoms (3) and anxiety (2) alone. This may indicate that CAD was a resultant condition from a more complex series of adverse events or influences, supporting our preliminary conception that CAD might represent more severe psychopathology or pathophysiology than other single disorders. Furthermore, it is equally important to note that the two more personality-related factors, namely, negative coping and perceived stress were the communal predictors for anxiety, depressive symptoms as well as CAD, indicating that these three conditions might have a common underlying pathogenesis mechanism; that is, an anxiety-prone personality accompanied by external stressors such as the COVID-19 related stress and lifestyle stressors such as staying up late/residential place might lead to depression and eventually CAD [17]. Finally, as COVID-19-related stress is the communal predictor for both depressive symptoms and CAD, it suggests that the pandemic does have a significant adverse impact on students' mental health.

In more specific terms, regarding negative coping, our study was able to identify it as the strongest predictor for all the above three conditions. Previous studies also have shown that coping was an important factor in both anxiety and depression in students [50, 51]. Individuals' behavioral and cognitive responses to difficulties were referred to as ‘coping’. The negative coping style represents an actual negative attitude, such as “Trying to forget the whole thing”, “Deciding to just wait and hope that time will change the current situation”, and “Hoping that something may happen which will change the current situation”. A negative coping style may lead to negative behaviors such as “Getting rid of the irritation caused by the problem by smoking, drinking alcohol, taking medicine, or eating” [29, 52]. Those negative attitudes and behaviors are evasive, which not only cannot solve the problems and challenges an individual is facing but also aggravate the feeling of being anxious, powerless, depressed, and down. Previous studies have shown that Cognitive Behavior Therapy (CBT) could reduce emotion-focused/ineffective ways of coping, which would be beneficial to improve anxiety and depression [53, 54]. In addition, previous studies showed that web-based [55] and video-based CBT [56] were effective. Thus, during the COVID-19 pandemic, universities could implement some web/video-based CBT programs to help students with the conditions. However, findings from our study with negative coping as the strongest predictor strongly suggest that, in addition to the above mentioned therapies, online programs aiming to cultivate appropriate coping styles in students may also be very important to achieve that end.

Consistent with previous studies, perceived stress in our study was found associated with CAD along with symptoms of anxiety and depression both during [12] and before [30, 31] the Covid-19 outbreak. In fact, perceived stress was one of the only two communal predictors for all three conditions, suggesting that successful interventions in reducing perceived stress may benefit all three mental health problems in students. In this regard, several strategies to reduce stress or increase the sense of control could be envisioned. Firstly, during the pandemic, it was found that restriction of physical activities could increase the level of stress. In addition, previous studies demonstrated that exceedingly sedentary behavior increased stress [57], and in a 6-year cohort study of university graduates in Spain, it was discovered that those who sat down for more than 42 h a week were 1.3 times more likely to experience stress, anxiety, and depression than those who sat down for only 10.5 h a week [58]. Restriction of physical activities may be unavoidable during the pandemic, but the university could encourage students to do some indoor sports to decrease the stress. Secondly, other concurrent psychological problems such as loneliness, uncertainty, and insecurity should be eliminated by, for example, providing online psychological consultations and class activities. Thirdly, a personalized curriculum schedule according to students’ situations to solve the problem of the time difference in different countries might help reduce the stress and burnout in students. Finally, it might be important to note that some previous studies have shown that perceived stress influenced anxiety and depressive symptoms via their mental adjustment [59, 60], suggesting that we might be able to reduce the level of stress through some auxiliary courses/lectures to educate the students how to correctly assess and deal with stress. Noticeably, mindfulness, especially mindfulness-based stress reduction(MBSR) was proved to be an effective program in many previous studies [61, 62], and online mindfulness programs had also been found feasible [63, 64]. Thus, online MBSR could be a practicable program for students to help release stress during the pandemic.

Regarding the stress associated with the Covid-19 pandemic, the finding that it was the predictor of depressive symptoms and CAD once again reminds us that, as the Covid-19 pandemic continues to be widespread throughout the world, there is still an urgent need to deal with the pandemic related to stress and minimize its adverse impact. Many intervention strategies, such as Solution Focused Brief Therapy [65], psychological counseling [66], and low-intensity cognitive behavior therapy [67], have proven to be effective to reduce depression during the pandemic. School administration may learn from these effective strategies, and develop their own suitable programs to help students cope with the stressors and reduce the impact of CAD during the pandemic.

The connection between staying up late and current residence with CAD was of no surprise because numerous studies have demonstrated how poor lifestyle choices like staying up late lead to mental health issues in students [68–70]. In this sense, more counseling and instructions may be needed to help students develop a healthy lifestyle. As to the predictor's current place of residence, we found that students residing outside of China in Asia and students staying in China both had a high level of CAD. The finding with students staying outside China was also not unexpected, because the majority of the study's participants were Indian students and India was experiencing an outbreak at the time the survey was conducted which could have had an adverse effect on the mental health of the students. But we were a little taken aback by the finding that students living in China also had elevated levels of CAD. We speculate that this might be due to the fact that they were away from home for a long period of time and were homesick or worried over their close relatives or friends during the pandemic. Nevertheless, this information reminded us that we should not underestimate the negative influence of COVID-19 on the mental well-being of international students residing in China.

However, a few findings of the present research did not endorse our hypothesis in that they revealed no significant associations between any of the three conditions and positive psychological variables like perceived social support, optimism, and resilience. To better understand how those variables influenced CAD and related disorders, further research is required.

### Limitations

There are a few limitations to this study. The cross-sectional design prevented the confirmation of the causal relationship. Longitudinal studies ought to be utilized in future research for addressing the relationship. In addition, a larger, the multi-center sample size is required to increase the data's representativeness. Nevertheless, amidst these drawbacks, our research has added new and helpful details about the mental health status of international medical students and offered potential solutions to lower or prevent CAD in the students.

### Comparison with prior work

The purpose of the present research is to determine the potential risk factors for CAD in international medical students studying in China and analyze the hypothetical sociodemographic and psychological variables that produced significant results. This study is significant as it provides important information on CAD in international medical students during the COVID-19 pandemic, which, according to our knowledge, hasn't been extensively studied before. Findings from this study have demonstrated that the roles of COVID-19 pandemic-related stress, staying up late, and current residence in the development of CAD, may be specific or related to the pandemic. These findings may help university authorities or educators focus their attention and efforts on helping international students in the best possible way. Additionally, the study's findings have served as a reminder that traditional mental health indicators like a poor coping style and perceived stress are indeed crucial indicators of students' mental health throughout the pandemic. Integration of such information into the intervention strategies for addressing the student mental health issues and development of educational programs to prevent CAD or related mental disorders might be effective and efficient.

## Conclusions

The incidence of anxiety or depression alone was moderate among international medical students in China. However, CAD was the most common and its prevalence was much higher than that of anxiety or depression alone, suggesting mental health problem in the students is relatively serious, and urgent attention is needed. In addition, the communal predictors for the three mental health problems were negative coping style and perceived stress, whereas Covid-19 pandemic-related stress was relatively specific for depression and CAD, and staying up late and current residential place were specific for CAD. The results imply that in order to assist international medical students in improving their mental health status during this COVID-19 pandemic, both conventional and situation-specific intervention measures should be adopted.

## Data Availability

The raw data supporting the conclusions of this article will be made available by the authors, without undue reservation. The datasets used and/or analysed during the current study available from the corresponding author on reasonable request.

## Abbreviations

CAD: Comorbid Anxiety and Depressive Symptoms
GAD-7: Generalized Anxiety Disorder Assessment
PHQ-9: Patient Health Questionnaire-9
SCSQ: Simplified Coping Style Questionnaire
PSS-10: Perceived Stress Scale-10
MSPSS: The Multidimensional Scale of Perceived Social Support
LOT-R: Revised Life Orientation Test
RS-14: Resilience Scale-14
